# A novel highly active and reusable carbon based platinum-ruthenium nanocatalyst for dimethylamine-borane dehydrogenation in water at room conditions

**DOI:** 10.1038/s41598-020-64046-9

**Published:** 2020-04-28

**Authors:** Yasar Karatas, Hilal Acidereli, Mehmet Gulcan, Fatih Sen

**Affiliations:** 1 Department of Chemistry, Faculty of Science, University of Van Yuzuncu Yıl, 65080 Tusba, Van Turkey; 2 Department of Biochemistry, Dumlupınar University, 43100 Kutahya, Turkey

**Keywords:** Energy storage, Hydrology, Catalysis, Energy science and technology

## Abstract

In this paper, we present platinum/ruthenium nanoparticles supported on Vulcan carbon (PtRu@VC) as a nanocatalyst for the dehydrogenation of dimethylamine-borane (DMAB) in aqueous solution under mild conditions. PtRu@VC nanocatalyst was fabricated using the alcohol-reduction techniques which is a facile and effective method. The prepared PtRu@VC nanocatalyst exhibited a good stabilization and an effective catalytic activity for hydrogen evolution from the DMAB dehydrogenation in water at room temperature. The composition of PtRu@VC nanocatalyst was investigated using different analytical techniques inductively coupled plasma optical emission spectroscopy (ICP-OES), transmission electron microscopy (TEM), high-resolution transmission electron microscopy (HR-TEM), powder X-ray diffraction (P-XRD) and X-ray photoelectron spectroscopy (XPS). A monodispersedPt/Ru metals distributions on VC (as supporting material) were revealed by TEM and HR-TEM analyses. The mean particle size of PtRu@VC nanocatalyst was found to be 3.15 ± 0.76 nm. XPS analysis for PtRu@VC nanocatalyst showed that almost Pt-Ru metals were found to be the metallic state. Catalytic experimental results showed that PtRu@VC nanocatalyst has a high catalytic activity with an excellent turn-over frequency (TOF_*initial*_) value of 14926.2 h^−1^ (248.77 min^−1^) in the dehydrogenation of DMAB in water at room temperature. Additionally, in the paper, we report some different kinetic data obtained from different experimental parameters of temperature, catalyst and substrate concentrations conducted for DMAB dehydrogenation in water catalyzed with PtRu@VC nanocatalyst.

## Introduction

Nanomaterials have been using in many different areas such as organic reactions, sensors, fuel cells, water treatment, hydrogen technologies, *etc*^1–10^. Nowadays, the most important problem to be overcome in hydrogen technologies is the availability of safe and efficient hydrogen storage materials^11,12^. Being in too low density is one of the most challenge issued to be overcome for the liquefaction and transportation of hydrogen at mild conditions. Therefore, the importance of chemicals to use effectively for hydrogen storage applications is increased day by day^4^. For this purpose, various chemicals have been tested to store and transport hydrogen. Several boron derivatives such as sodium borohydride (NaBH_4_), hydrazine (N_2_H_4_), ammonia-borane (NH_3_BH_3_) have been investigated for this purpose. Additionally, alkyls, some magnesium/calcium borohydrides, mesoporous/microporous materials have been also applied as solid hydrogen storage materials^7,13–24^. The chemical-based boron derivatives and nitrogen have many more advantages compared to these chemicals due to being a high storage capacity of hydrogen. These boron derivatives and nitrogen-based chemicals exhibit unique charge/discharge feature^25–28^. The material-based boron and nitrogen have features like porous that facilitate the hydrogen release at the room conditions. DMAB is a boron-based chemical, one of amine-borane derivate, has a (CH_3_)_2_NHBH_3_ chemical formula. Hitherto, several studies^29,30^ have been conducted relevantto DMAB using a suitable catalyst at mild conditions. The results of studies revealed that DMAB can act as a hydrogen source through its hydrogenous reaction in case of using a suitable catalyst at the mild condition^31–34^.

The water solubility of DMAB is high at room temperature that is an advantage for DMAB hydraulic dehydrogenation^35^. DMAB is present in a stable form at mild conditions and its hydraulic dehydrogenationcan not occur spontaneously. In the case of using 1 mol DMAB results in 3 mol hydrogen gas through the hydrolytic dehydrogenation reaction of DMAB with a suitable catalyst at room conditions. However, this reaction results in 1 mol hydrogen gas, using 1 mol DMAB, by using organic solvents at the same conditions^35–38^. Table 1 shows experimental results about some nanocatalyststhat have been applied to DMAB dehydrogenation in the water at mild conditions. Those of nearly all catalysts are the heterogeneous catalyst, not have full of active sites and their number of active atoms is limited. Having not enough active atom on the surface catalyst results in reduced catalytic activity^39–41^. Literature studies showed that the catalysts based metal nanoparticles can be dispersed aquatic solution that increases the number of active atoms, several active sides on the surface catalysts and that results in the increased catalytic activity of DMAB at requested medium conditions^42–49^. Therefore, effectively using metal-based nanomaterials as catalysts for the DMAB dehydrogenation reaction have been proven and the new metal-based catalyst must be developed for DMAB dehydrogenation.

**Table 1:** Some experimental TOF_*initial*_ values of catalysts used for DMAB dehydrogenation in water.

Catalyst	TOF_*initial*_(h^−1^)	Reference
PtRu@VC	14926.2	This study
NiSO_4_/Na_2_WO_4_	4.2	^42^
Carbon supported Pd	30	^37^
NiSO_4_/ KReO_4_	3.6	^42^
NiSO_4_/Na_2_MoO_4_	2.8	^42,43^
Ru NPS	500	^62^
Rh NPs@MWCNT	3010.5	^63^

mol H_2_/(mol catalyst × h).

**Scheme 1:**
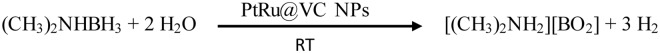
The schematic representation of DMAB dehydrogenation in an aquatic medium.

In this paper, we introduce a new platinum/ruthenium nanoparticles decorated on Vulcan carbon (PtRu@VC) as a nanocatalyst for DMAB dehydrogenation in water. In the paper, the chemical composition of PtRu@VC nanocatalystwas highlighted by advanced analytical techniques including transmission electron microscopy (TEM), high-resolution transmission electron microscopy (HR-TEM), powder X-ray diffraction (P-XRD) and X-ray photoelectron spectroscopy (XPS). Further, some experimental studies and their kinetic findings for the prepared nanocatalyst in DMAB dehydrogenation in water were performed. The experimental results were shown the good distribution of PtRu alloy (with a metallic form of Pt(0) and Ru(0)) nanoparticles on VC support material. The schematic representation of DMAB dehydrogenation in an aquatic medium is given in Scheme 1.

The surface area of the Vulcan carbon is about 250 m^2^ g^−1^. This very large surface area provides more active sites on it. In addition, the electrical conductivity of VC approximately 2.77 Scm^−1^ makes it easy to electron transfer at room temperature. These properties make Vulcan carbon a favorable support material in DMAB dehydrogenation. Another reason why vulcanic carbon is preferred in DMAB dehydrogenation is its high stability. Moreover, PtRu bimetallic nanoparticles showed a very good distribution on Vulcan carbon^50,51^. As results of the conducted experimental findings and calculation of kinetic studies, Vulcan carbon-supported Pt-Ru nanoparticles can catalyze in the dehydrogenation of DMAB with a record activity (initial turn-over frequency, TOF_*initial*_ = 14926.2 h^−1^ (248.77 min^−1^)) at high conversion (>99%) under mild conditions (at 298 K andunder air). This new superior nanocatalyst enables facile catalyst recovery and very high stability against agglomeration, and leaching, which make it highly recycle catalyst (retains ~64% activity and >99% conversion at the end of 10^th^ catalytic recycle) in the dehydrogenation of DMAB in water under the room conditions.

## Experimental procedure

### The fabrication of Pt@VC, Ru@VC and PtRu@VC nanocatalysts

The fabrication of platinum/ruthenium nanoparticles supported on Vulcan carbon bimetallic nanocatalyst was carried out using an alcohol reduction process as described elsewhere^52^. Briefly, a solution containing 50 mL water/ethyl alcohol mixture, 2.5 mmol Vulcan carbon, and 0.25 mmolK_2_PtCl_4_ and 0.25 mmol RuCl_3∙_3H_2_O was prepared. The resulting solution was refluxed at 90 °C for 2 h. On the other hand, in two separate experiments, 0.25 mmol solution (Pt from K_2_PtCl_4_ and Ru from RuCl_3∙_3H_2_O) is mixed with Vulcan carbon. The resulting solutions were refluxed at 90 °C for 2 h. The obtained PtRu@VC, Pt@VC and Ru@VC nanocatalysts were filtered and washed several times with water and stored for further usages.

### Experimental and kinetic studies of DMAB dehydrogenation in water catalyzed by PtRu@VC nanocatalyst

Three experimental parameters were performed to detect the effect of PtRu@VC nanocatalystfor DMAB dehydrogenation in water. These parameters were temperature, PtRu@VC nanocatalystand substrate (DMAB) concentrations. Temperature studies were performed with different temperatures in the range of 293–308 K using a 5 mL mixture including PtRu@VC nanocatalyst(25 mg, 4.96 µmol, 0.992 mM) and DMAB substrate (15.2 mg, 0.25 mmol; 50 mM). Substrate studies were carried out to detect the effect of DMAB concentration in the range of 25–100 mM containing PtRu@VC nanocatalyst(25 mg, 4.96 µmol, 0.992 mM) at room temperature. The catalyst concentration studies were conducted with different PtRu@VC nanocatalystconcentrations in the range of 0.59–1.19 mM containing DMAB substrate (15.2 mg, 0.25 mmol; 50 mM) at room temperature. The temperature experimental results and Eyring/Arrhenius plots were used to calculate kinetic calculations of DMAB dehydrogenation in water including PtRu@VC nanocatalyst^53,54^.

## Results and discussion

### Highlighting chemical and structural compositions of PtRu@VC nanocatalyst

Devices used for characterization are provided in Supporting Information. To prepare PtRu@VCbimetallic and Pt@VC, Ru@VC monometallic nanocatalysts, the alcohol/reduction technique was used using Vulcan carbon as a support material at room conditions. The molar composition of the prepared PtRu@VC nanocatalyst was found to be Pt_0.40_Ru_0.60_ (1.56% wtPt and 1.20% wtRu loadings correspond to 7.99 μmol Pt and 11.87 μmol Ru) by ICP-OES. This ICP-OES value was used to calculate all the nanocatalyst concentrations in the kinetic studies. To find the effects of the support material on the formation of nanocatalyst, firstly, a solution was prepared non-including VC and an agglomeration was observed in the resulting solution. This solution including Pt^2+^/Ru^3+^and chloride (Cl^−^) ions. It can be said that the only chloride ion isn’t enough to stabilize these nanoparticles. The same solution but including VC was prepared and the stabilization of the nanoparticles was achieved not seen any agglomeration and precipitation. That results reveal the stabilization effects of VC as a stabilizer materials. We can say PtRu alloy metals were stabilized by VC support materials. The reduction Pt(II) and Ru(III) ions into Pt(0) and Ru(0) metallic states were achieved by using DMAB as a reducing agent. The changing solution color from light brown to dark brown showed the reduction of these ions to their metallic state.

**Figure 1:**
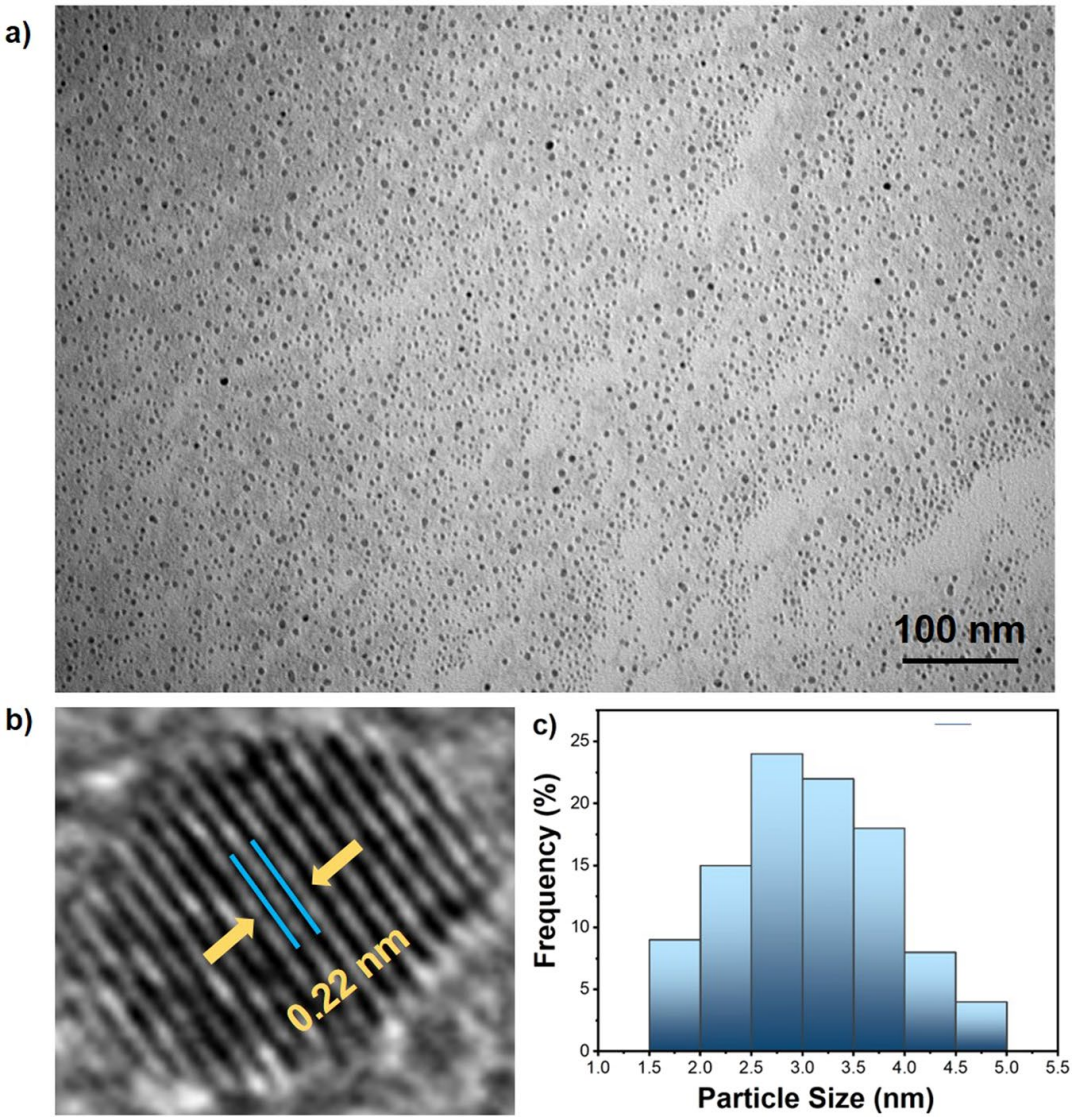
(**a**) TEM image in 100 nm scale (**b**) HR-TEM, (**c**) the mean particle size of PtRu@VC nanocatalyst.

The chemical dispersions of PtRu alloy nanoparticles on VC support material were investigated by TEM analysis as given in Fig. 1(a,b). As seen in Fig. 1(a), PtRu nanoparticles showed a homogeneous distribution on the VC support material and there was no aggregation. Most of the nanoparticles are spherical. The atomic lattice fringe was determined as 0.22 nm from HR-TEM in Fig. 1(b). The mean particle size of PtRu@VC nanocatalyst was calculated by counting almost 300 particles and the mean particles were found to be 3.15 ± 0.76 nm as seen in Fig. 1(c).

**Figure 2:**
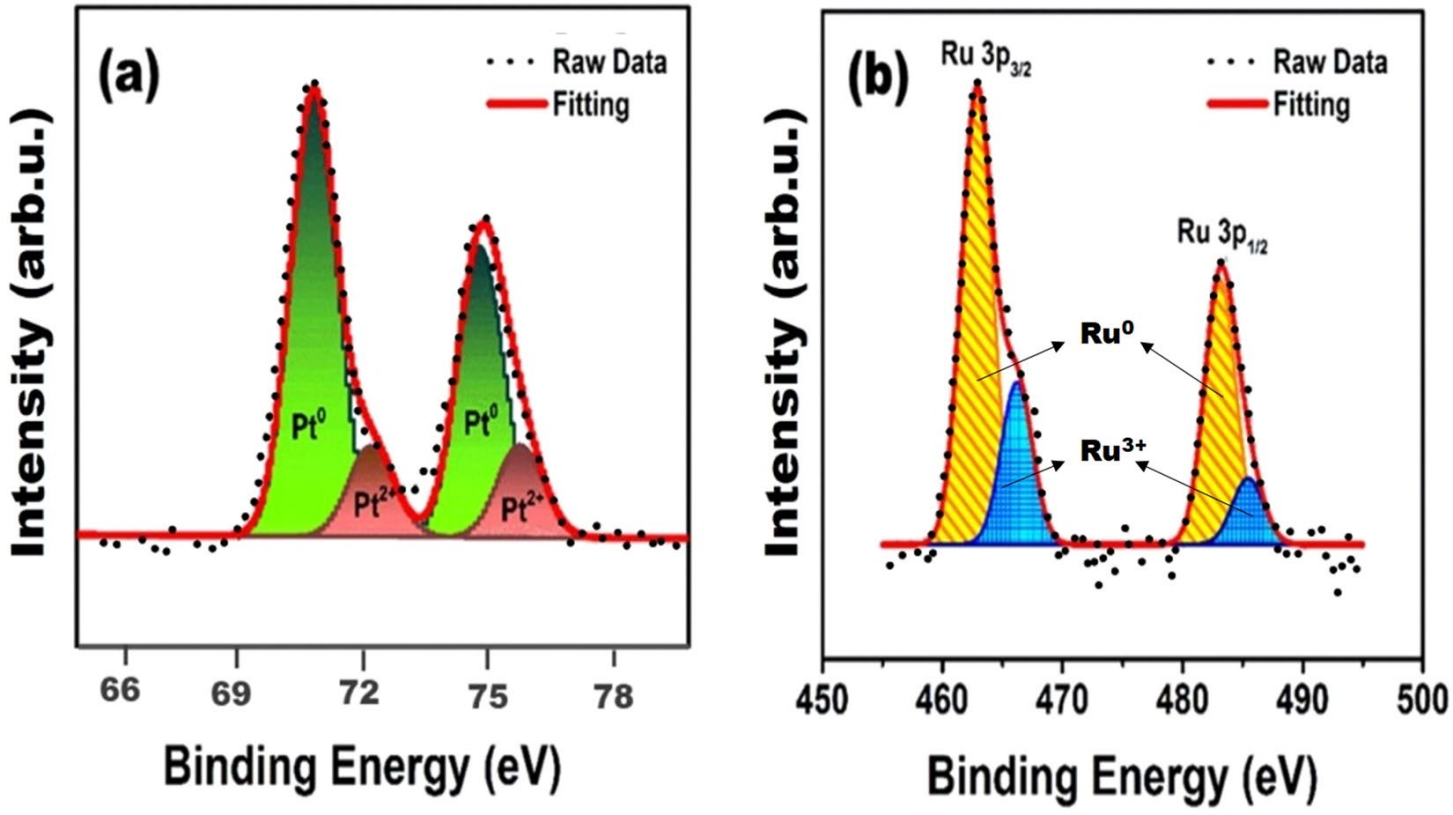
(**a**) Pt 4f and (**b**) Ru 3p region high-resolution XPS analysis for PtRu@VC nanocatalyst.

The metallic formation of Pt (0) and Ru (0) nanoparticles, their oxidation states and surface compositions were also investigated by applying XPS analysis at room conditions. The XPS analysis results are given in Fig. 2(a,b). As seen in Fig. 2, two evidence peaks were obtained at 71.0–74.5 eV and 462.8–483.2 eV compatible with Pt (0) 4f_7/2_-4f_5/2_ and Ru (0) 3p_3/2_-3p_1/2_^55,56^. And some peaks belong to Pt (II) and Ru (III) at 72.3–75.9 eV and 466.2–485.3 eV seen in Fig. 2(a,b) were observed. These peaks show some formations oxidic Pt_2_O_3_ and RuO_2_ on the surface of PtRu@VC nanocatalyst during the sample preparation process for XPS analysis^55,56^.

**Figure 3:**
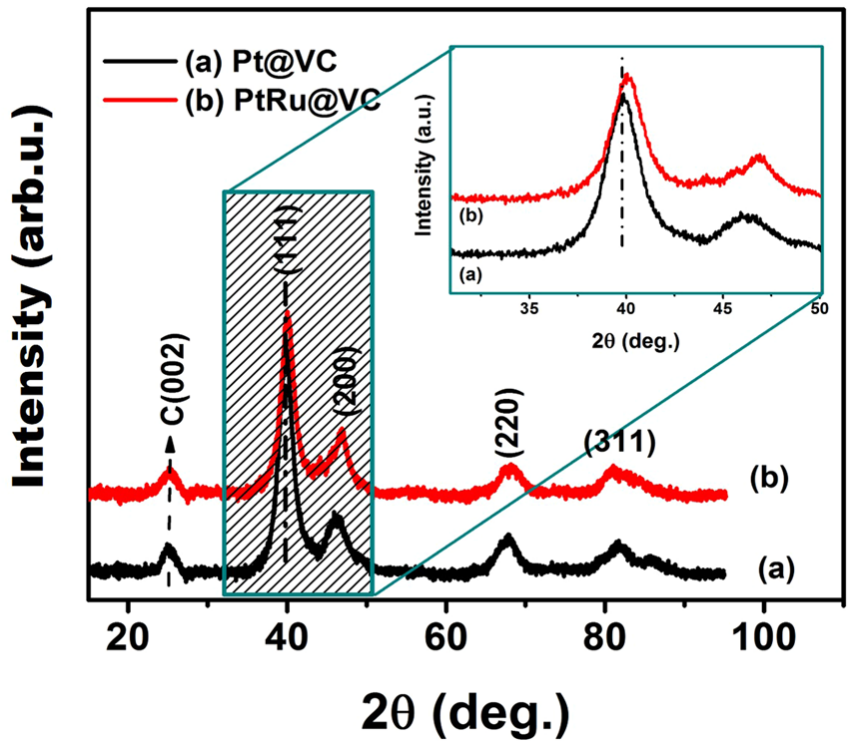
XRD patterns of Pt@VC and PtRu@VC nanocatalysts.

In the literature, the atomic lattice parameter of pure Ptnanometals is 3.925 Å^57^. The lattice parameter of the PtRu @ VC nanocatalyst was calculated as 3.453 Å with the Bragg equation. The reason for this decrease is that Pt combines with the smaller Ru than itself and forms an alloy. The crystal structure and morphology for PtRu@VC nanocatalyst were investigated using P-XRD method. The P-XRD analysis results are given in Fig. 3. P-XRD pattern of Pt@VCmaterial the peaks observed at 2θ degrees of 25.3°, 39.82°, 46.19°, 67.94°, 81.82° are related to C (002), Pt (111), (200), (220), and (311) planes, respectively. Similarly, in P-XRDdiffractogram of PtRu@VC nanocatalyst, the peaks detected at 25.78°, 40.11°, 47.10°, 68.41°, 81.35° are corresponding to C (002), Pt (111), (200), (220), (311) planes, respectively. The slight shift on the P-XRD patterns indicated that Pt and Ru are in alloy form and particle size change due to the smaller Ru atoms. In addition, the average crystal particle size was calculated as 3.15 nm by Scherrer equation and it was found to be compatible with TEM results.

### Catalytic experiments and kinetic studies of DMAB dehydrogenation in water using PtRu@VC nanocatalyst

Several experimental studies were performed to detect the effects of PtRu@VC nanocatalyst in DMAB dehydrogenation in water. The experimental set up used in the studies was used as given in our previous study^6^. The catalytic activities of PtRu@VC nanocatalysttogether with its monometallic (Pt@VC and Ru@VC) counterparts (in equimolar total metal concentrations) were investigated the catalytic dehydrogenation of DMAB in the water at room temperature and their results are presented in Fig. S1(a). PtRu@VC nanocatalyst provides a better activity than those of monometallic nanocatalysts prepared by the same method. In addition, the best molar composition for bimetallic PtRunanocatalyst was found to be 0.40:0.60 (Pt:Ru) in terms of catalytic activity and total conversion (Fig. S1(b)).

**Figure 4:**
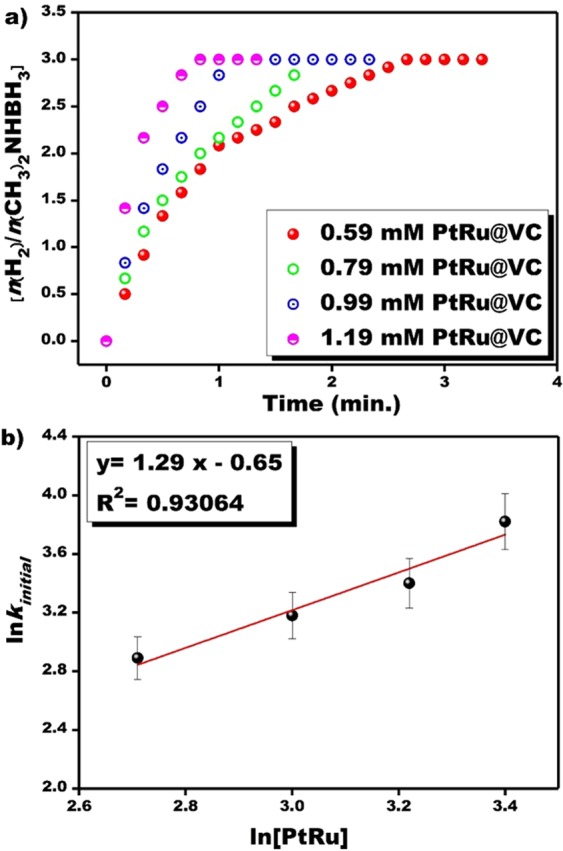
(**a**) The plot of hydrogen volume obtained from the experiments conducted with different PtRu@VC nanocatalyst concentrations (**b**), the plot of ln[PtRu] versus ln(*k*_*initial*_) at room conditions.

The produced hydrogen gas volume obtained from experiments conducted with the different PtRu@VC nanocatalyst at room temperature is given in Fig. 4(a). As seen in Fig. 4, the hydrogen release was started not observed any induction time, and the catalytic dehydrogenation of DMAB in water catalyzed with PtRu@VC nanocatalyst was finished in a short time under mild conditions. The released H_2_ volume was increased with increased PtRu@VC nanocatalyst concentrations. After these experimental studies, a plot of ln[PtRu] versus ln (*k*_*initial*_) was drawn using data obtained from Fig. 4(a), as seen in Fig. 4(b), the drawn plot is nearly a linear graph with R^2^ = 0.93064 value. From Fig. 4(a,b), it was determined that the degree of reaction due to the catalyst was 1.29. The reaction rate obtained kinetic data of the PtRu@VC nanocatalyst concentration studies for DMAB dehydrogenation in water was found to be compatible with the first-order model (Fig. 4(b)).

**Figure 5:**
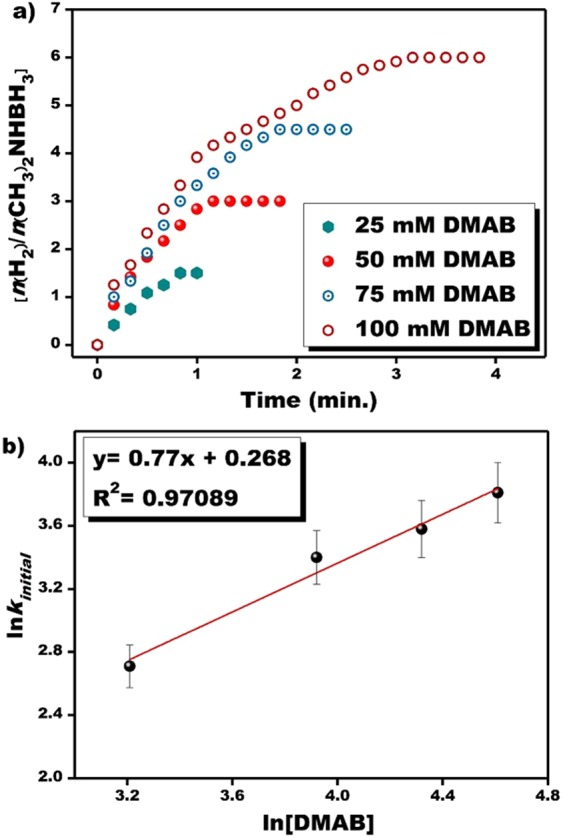
(**a**) The plot of hydrogen volume obtained from the experiments conducted with different DMAB substrate concentrations, (**b**) the plot of ln[DMAB] versus ln(*k*_*initial*_) at room conditions.

Similar experiments were applied using the different DMAB concentrations in a range of 25–100 mM under the common experimental parameters and the results of these experiments are given in Fig. 5(a). The experimental data obtained from Fig. 5(a) were used to draw the plot of ln[PtRu] versus ln (*k*_*initial*_) and the drawn plot was found to be a linear graph as seen in Fig. 5(b). The regression coefficient of Fig. 5(b) was determined to be R^2^ = 0.97089. The evaluations of these data showed that the catalytic dehydrogenation of DMAB catalyzed with PtRu@VC nanocatalyst conducted with different DMAB concentrations was compatible with the 0.77 order-equation (Fig. 5(b)).

**Figure 6:**
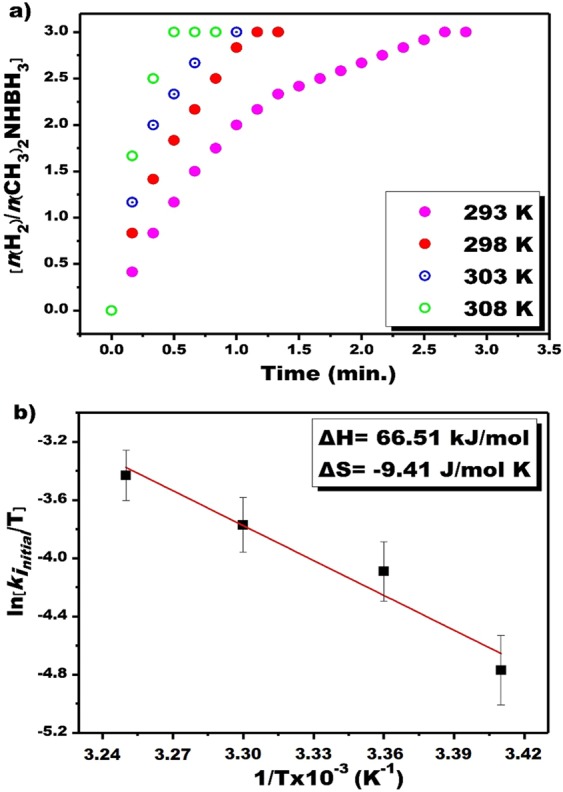
(**a**) The plot of hydrogen volume obtained from the experiments conducted at different temperatures, (**b**) the plot of Eyring at room conditions.

The experimental results obtained from the different temperatures in a range of 298–308 K are given in Fig. 6. The hydrogen release was initiated upon the catalytic reaction started unseen any induction time and with the increased temperature the volume of hydrogen gas has been increased as seen in Fig. 6. TOF_*initial*_ value for PtRu@VC nanocatalyst in DMAB dehydrogenation in water was determined as 14926.2 h^−1^ (248.77 min^−1^) that value implies this catalyst is very effective in the catalytic dehydrogenation of DMAB in water under room conditions compared the other tested catalyst for this important catalytic reaction (details for the calculation of initial TOF_*initial*_ value is given in the Supporting Information). When calculating the initial TOF_*initial*_ value, the number of moles of the nanocatalyst was determined by considering ICP-OES value. When calculating catalyst concentrations, not only metal atoms on the surface but also all-metal atoms (both surface and interior) were taken into account. Therefore, the concentration expression used herein should be considered as a measure of percent metal loading rather than a real concentration using surface atoms.

Using experimental results, kinetic data, Eyring and Arrhenius equations, activation parameters of the catalytic dehydrogenation of DMAB in water catalyzed with PtRu@VC nanocatalyst were calculated. The results are given in Fig. 6 ^58,59^. The activation energy (Ea), activation enthalpy (ΔH) and activation entropy (ΔS) of catalytic dehydrogenation of DMAB in water were found as 37.36 kJ/mol, 66.51 kJ/mol and −9.41 J/(mol × K), respectively. The ln (k_*initial*_/T) graph corresponding to 1/T drawn using the experimental data in Fig. 6(b) is a linear graph (R^2^ = 0.95860). Assuming that the activation parameters calculated from the macroscopic kinetic data given above are relevant to the most critical activation step in the decomposition of DMAB, one can argue that the positive magnitude of the apparent activation enthalpy and the negative value of apparent activation entropy imply the presence of an associative mechanism at the transition state.

Mercury poisoning test was also performed to determine the catalytic activity of catalytic dehydrogenation of DMAB in the study. For this purpose, mercury was added to the DMAB substrate and a decrease in DMAB catalytic reaction performance was observed. Mercury poisoning test results showed that PtRu@VC nanocatalyst is a water-dispersible and heterogeneous catalyst for DMAB dehydrogenation in water^60,61^.

**Figure 7:**
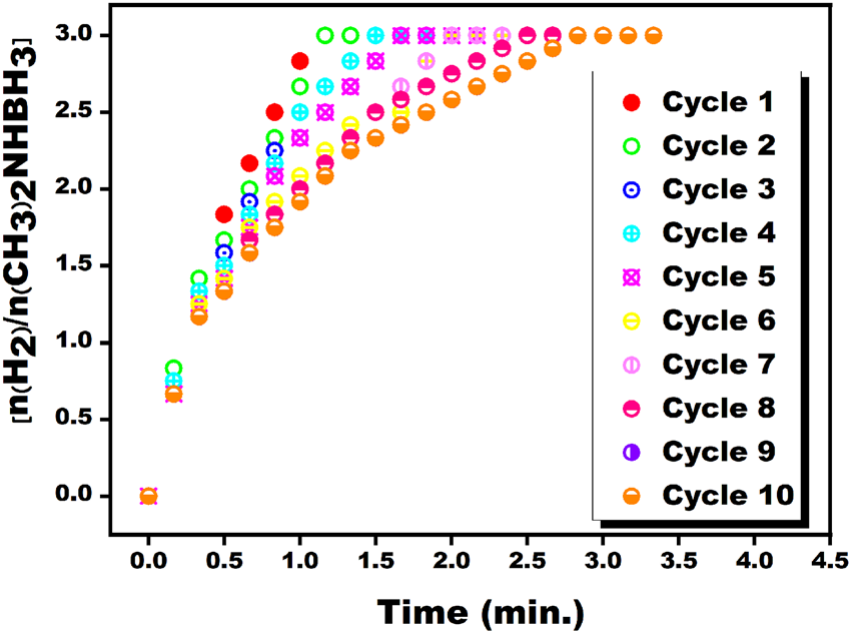
The experimental results of the recyclability of PtRu@VC nanocatalyst in DMAB dehydrogenation in the water at room conditions.

The catalytic life of the PtRu@VC nanocatalyst was tested by recycling experiments. When only the solution containing mass PtRunanometals was added to the solution medium containing the DMAB substrate, low catalytic activity was observed. The catalytic recycle experiments were tested by 10 runs to determine the stability of PtRu@VC nanocatalyst in DMAB dehydrogenation in the water at room conditions. A simple catalytic recycle experiment can be summarized as follows; At the end of an experiment whose reaction was finished, an amount of DMAB substrate equal to the amount of DMAB initially taken was added to the reaction medium and the DMAB catalytic reaction was re-started. This procedure was repeated ten times. The amount of H_2_ gas obtained by these experiments for the PtRu@VC nanocatalyst was obtained as a percentage. These values found as given in Fig. 7 that calculated as the reusability of the PtRu@VC nanocatalyst. The recycle test results showed that the catalytic activity of the PtRu@VC nanocatalyst retained almost 64% at the end of the 10^th^ run. With these results, it was concluded that PtRu@VC nanocatalyst obtained for DMAB dehydrogenation reaction in the aqueous medium had a complementary effect. Table 1 shows some catalysts and their TOF_*initial*_ values that are tried for DMAB dehydrogenation in water. Compared to these catalysts, the PtRu@VC nanocatalyst was found to have the highest TOF_*initial*_ value among other heterogeneous catalysts. It can be said that the VC substrate is stabilized by PtRu alloy nanometals and the increase in the number of active sites formed on the surface of the catalyst causes this result.

## Conclusions

In this study, PtRu@VC nanocatalyst was prepared by a practical method of alcohol reduction techniques using K_2_PtCl_4_, RuCl_3_ ∙ 3H_2_O, and VC. The morphological and surface distribution structure of PtRu@VC nanocatalyst were elucidated using various advanced analytical techniques such as TEM, XPS, and XRD. The mean particle size of the obtained PtRu@VC nanocatalyst was calculated as 3.15 ± 0.76 nmby TEM analyzes. Catalytic experiments have shown that the PtRu@VC nanocatalyst is highly effective in the DMAB dehydrogenation in water, even at low temperatures. The catalytic activity of the PtRu@VC nanocatalyst was compared with some catalysts found in the literature and found to have a high TOF_*initial*_value (248.77 min^−1^ or 14926.2 h^−1^) at high conversion (>99%) compared to other catalysts tested at room conditions. Mercury poisoning test results showed that PtRu@VC nanocatalyst is a water-dispersible and heterogeneous catalyst for DMAB dehydrogenation in water. The activation energy (Ea), activation enthalpy (ΔH) and activation entropy (ΔS) of DMAB dehydrogenation in water were calculated as 37.36 kJ/mol, 66.51 kJ/mol and −9.41 J/(mol × K), respectively. As a result, the resulting PtRu@VC nanocatalystcan be used with high catalytic efficiency and high stability (~64% activity and >99% conversion at the end of 10^th^ catalytic recycle) for DMAB dehydrogenation in water. Also, it has been found that the PtRu@VC nanocatalyst can be used as a high potential catalyst for future fuel cells and other energy sources.

## Supplementary information

Supplementary Information.
