# Supplementary material for: A novel highly active and reusable carbon based platinum-ruthenium nanocatalyst for dimethylamine-borane dehydrogenation in water at room conditions
Source: Sci Rep. 2020 Apr 28;10:7149. doi: 10.1038/s41598-020-64046-9 (PMC7188795; doi:10.1038/s41598-020-64046-9)
Supplement: Supplementary file 1 — Supplementary Information. [file 41598_2020_64046_MOESM1_ESM.docx]

**A novel highly active and reusable carbon based platinum-ruthenium nanocatalyst for dimethylamine-borane dehydrogenation in water at room conditions**

**(SUPPORTING INFORMATION)**

Yasar Karatas^1^, Hilal Acidereli^2^, Mehmet Gulcan*^1^, Fatih Sen*^2^

*^1^Department of Chemistry, Faculty of Science, University of Van Yuzuncu Yıl, 65080, Tusba, Van, Turkey;^2^Department of Biochemistry, Dumlupınar University, 43100, Kutahya, Turkey*

*Corresponding authors E-mail: [mehmetgulcan65@gmail.com](mailto:mehmetgulcan65@gmail.com), [fatih.sen@dpu.edu.tr](mailto:fatih.sen@dpu.edu.tr)

***Calculation Method for Initial TOF Value:***

The calculation method for the *initial* TOF value 14926.2 h^-1^ (248.77 min^-1^) in the dehydrogenation of DMAB in water at room temperature is given below step by step.

1. The amount of nanocatalyst used for each test is 25 mg;
2. According to the ICP-OES data, (ICP-OES: PtRu@VC nanocatalyst contain % 1.56 Pt, % 1.20 Ru)**.** 25 mg of nanocatalyst used for each catalytic experiment were found to be 4.96 µmol (4.96 × 10^-6^ mole)
3. In order to calculate the initial TOF value, it was determined that the volume of H_2_ in which 20% of the substrate was converted to product and how long it took place.
4. From the following figure in the presence of 4.96 × 10^-6^ mole PtRu@VC nanocatalyst at 298 K, 20% conversion of DMAB to H_2_ (3.64 mL) at ~0.121 min.
5. The volume of 1 mol of gas at room conditions is 24.45 L (24450 mL)

*initial* TOF = [mole of product] / [mole of catalyst × time]

*initial* TOF = [3.64 mL/24450 mL]/[( 4.96 × 10^-6^ mole) × (0.121 min.)]

*initial* TOF ~ 248.77 min^-1^ (14926.2 h^-1^)

***Equipments***

Sample preparation was achieved by dropping the PtRu@VC nanocatalyst solution on a carbon-coated copper grid for TEM and HRTEM analysis. The JEOL 200 kV was employed for obtaining TEM images.

X-ray diffraction (XRD) analysis was performed using an XRD device having CuKα radiation and operating with 45 kV and a 2θ range of 20–90° (Panalytical Emperian, Turkey).

The electronic structure of PtRu@VC nanocatalyst was investigated using X-ray photoelectron spectroscopy (XPS, PHI 5000 Versa Probe). XPS spectrum was poltted using OriginLab Pro 2019 software. Shirley type background was applied prior to fitting. The fitting of the spectrum was achieved with Gaussian function.








**Fig. S1.** (a) The plot of the volume of the generated H_2_ gas versus time for monometallic (Pt@VC, Ru@VC) and bimetallic (PtRu@VC) nanocatalysts in DMAB dehydrogenation in water under the room conditions (in all 25 mg nanocatalyst, 15.2 mg DMAB in 5.0 mL aqueous solution) at 298 K. (b) The plot of hydrogen volume obtained from the experiments conducted with different molar ratios of PtRu@VC nanocatalyst

***The catalytic mechanism:***
